# Closing the Gap between Experiment and Simulation—A Holistic Study on the Complexation of Small Interfering RNAs with Polyethylenimine

**DOI:** 10.1021/acs.molpharmaceut.3c00747

**Published:** 2024-02-19

**Authors:** Jonas Binder, Joshua Winkeljann, Katharina Steinegger, Lara Trnovec, Daria Orekhova, Jonas Zähringer, Andreas Hörner, Valentin Fell, Philip Tinnefeld, Benjamin Winkeljann, Wolfgang Frieß, Olivia M. Merkel

**Affiliations:** Faculty for Chemistry and Pharmacy, Ludwig-Maximilians-Universität München, 81377 München, Germany; Center for NanoScience (CeNS), Ludwig-Maximilians-Universität München, 80799 München, Germany; Chair of Experimental Physics I, University of Augsburg, 86519 Augsburg, Germany; Faculty for Chemistry and Pharmacy, Ludwig-Maximilians-Universität München, 81377 München, Germany; Faculty for Chemistry and Pharmacy, Ludwig-Maximilians-Universität München, 81377 München, Germany; Center for NanoScience (CeNS), Ludwig-Maximilians-Universität München, 80799 München, Germany; Chair of Experimental Physics I, University of Augsburg, 86519 Augsburg, Germany; Faculty for Chemistry and Pharmacy, Ludwig-Maximilians-Universität München, 81377 München, Germany; Faculty for Chemistry and Pharmacy, Ludwig-Maximilians-Universität München, 81377 München, Germany; Center for NanoScience (CeNS), Ludwig-Maximilians-Universität München, 80799 München, Germany; Faculty for Chemistry and Pharmacy, Ludwig-Maximilians-Universität München, 81377 München, Germany; Faculty for Chemistry and Pharmacy, Ludwig-Maximilians-Universität München, 81377 München, Germany; Faculty for Chemistry and Pharmacy, Ludwig-Maximilians-Universität München, 81377 München, Germany; Center for NanoScience (CeNS), Ludwig-Maximilians-Universität München, 80799 München, Germany

**Keywords:** polyplex, RNA interference, rationale design, Martini 3

## Abstract

Rational design is pivotal in the modern development of nucleic acid nanocarrier systems. With the rising prominence of polymeric materials as alternatives to lipid-based carriers, understanding their structure−function relationships becomes paramount. Here, we introduce a newly developed coarse-grained model of polyethylenimine (PEI) based on the Martini 3 force field. This model facilitates molecular dynamics simulations of true-sized PEI molecules, exemplified by molecules with molecular weights of 1.3, 5, 10, and 25 kDa, with degrees of branching between 50.0 and 61.5%. We employed this model to investigate the thermodynamics of small interfering RNA (siRNA) complexation with PEI. Our simulations underscore the pivotal role of electrostatic interactions in the complexation process. Thermodynamic analyses revealed a stronger binding affinity with increased protonation, notably in acidic (endosomal) pH, compared to neutral conditions. Furthermore, the molecular weight of PEI was found to be a critical determinant of binding dynamics: smaller PEI molecules closely enveloped the siRNA, whereas larger ones extended outward, facilitating the formation of complexes with multiple RNA molecules. Experimental validations, encompassing isothermal titration calorimetry and single-molecule fluorescence spectroscopy, aligned well with our computational predictions. Our findings not only validate the fidelity of our PEI model but also accentuate the importance of *in silico* data in the rational design of polymeric drug carriers. The synergy between computational predictions and experimental validations, as showcased here, signals a refined and precise approach to drug carrier design.

**Figure:**
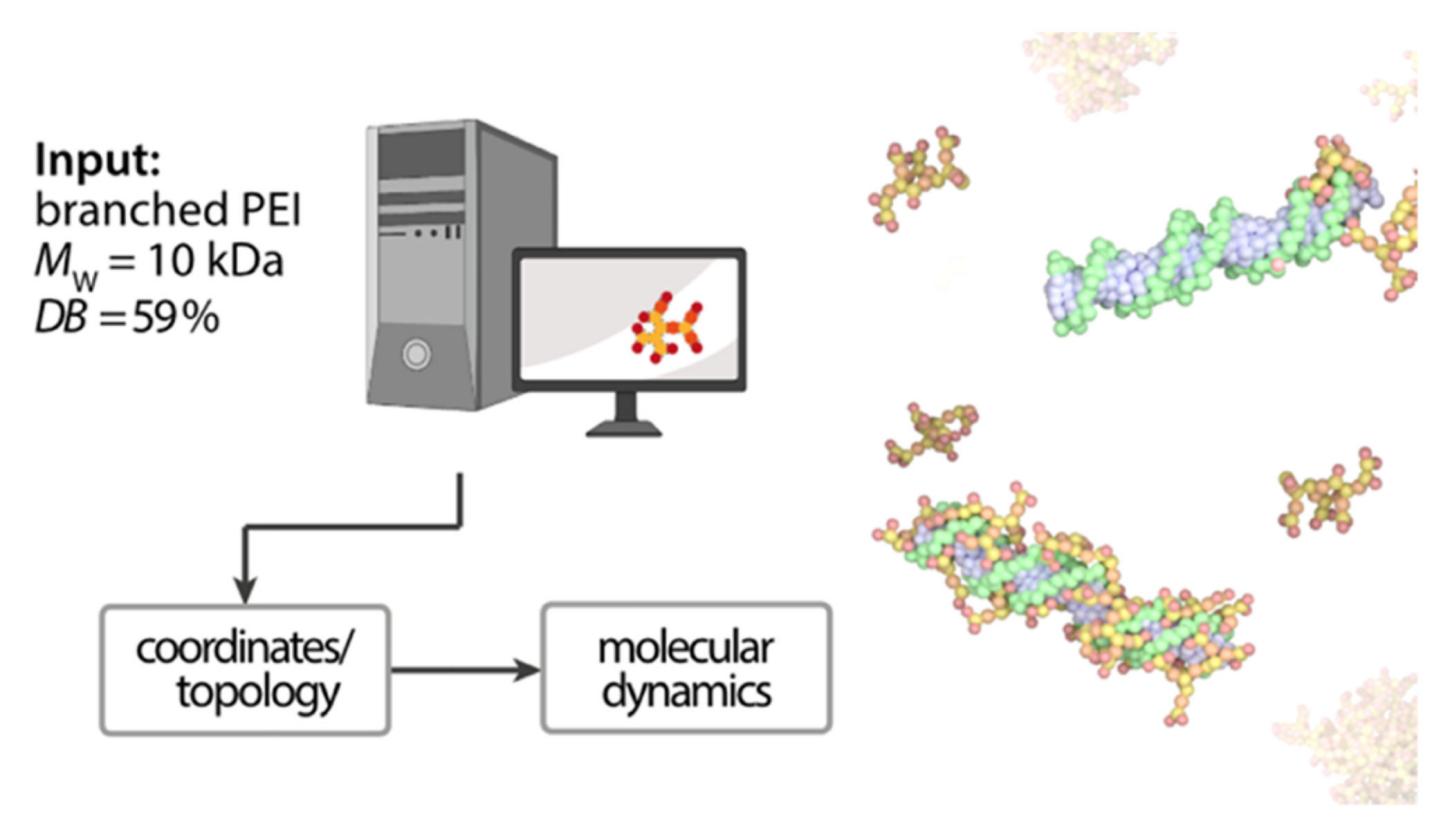

## 1 Introduction

Over the past years, RNA-based therapies have gained attention from not only researchers and pharmaceutical companies but also capital providers and the general public. This change was boosted by the successful approval of *Patisiran*, an RNA interference-based drug, in 2018.^1−3^ The first use of messenger (m)RNA as a vaccine to prevent SARS-CoV-2 infections, set another hallmark.^4,5^ As of January 2024, more than 20 RNA-based drugs have been authorized for inpatient use, and hundreds of new candidates are in the clinical pipelines.^6−8^ The success of RNA therapeutics can particularly be attributed to their promise to target previously undruggable disease causes.

All nucleic acids face the obstacles of a short *in vivo* half-life and poor cellular uptake. To overcome these limitations, RNA therapeutics in clinical use, as well as those in commercial pipelines and research, either make use of carrier systems or specific modification and conjugation techniques. Carrier systems can be categorized as viral or nonviral vectors. In particular, the nonviral lipid nanoparticles (LNPs) made enormous clinical progress in the past years despite their tendency to accumulate in liver tissue^9^ and a low API load, e.g., 4% w/w mRNA/lipid in *Comirnaty*.^10^

Polymeric cationic carrier systems have emerged as nonviral vector alternatives with a backlog in clinical stages. Besides their advantages when it comes to loading capacity where a drug-to-excipient ratio of 1 or higher can be achieved,^11−13^ polymeric systems offer tremendous tunability and easy scalability of production processes. Polyethylenimine (PEI), polyamidoamine (PAMAM), polylysine (PLL), poly(*β*-amino ester) (PBAE), or spermines are among the most frequently studied materials.^14−19^ Modified or block copolymers became popular to add extra features such as improved stability, targeting with ligands, or shielding.^20−26^ PEI as one of the first and most commonly used polycations for RNA delivery shows high loading capacity and transfection efficiency *in vitro*,^27,28^ but its high cation density and resulting toxicity^29,30^ impedes the translation of PEI into application in human.

Polycations, whether it be PEI or others, encapsulate nucleic acids by means of Coulombic forces, forming so-called polyelectrolyte or short polyplexes. The formation of polyplexes comes with a reduction in enthalpy *H* and a loss in the complexes’ entropy *S*. An overall decrease in Gibbs free energy *G* ensures thermodynamic equilibrium of such polyelectrolyte complexes.^31−33^ As electrostatic interactions are strongly affected by environmental conditions, especially ion concentrations, it is necessary to evaluate the biological route of the carrier. Upon administration, the drug delivery system will encounter a neutral pH in the extracellular environment, where salt, proteins, and enzymes are present, regardless of the chosen route. The complex itself must remain physically stable in this environment while safeguarding the RNA cargo from the degrading environment. *In vivo* it has been observed that RNA was released prematurely long before the target was reached.^34^

Once at the target cell surface, the carrier must facilitate the cellular uptake of the cargo. After internalization, the endosome presents another significant obstacle for the nucleic acid to become therapeutically active. To escape the endosome, various mechanisms have been proposed for polycations such as PEI, most of which involve the polymeric carrier being more heavily protonated when the pH drops along the endosomal degradation pathway.^35^ Similar mechanisms are described for lipid-based carriers, with all approved LNP formulations containing ionizable lipids that change their net charge from neutral to cationic when the pH falls below ≈6 to facilitate endosomal escape.^36^ This change in protonation, however, for polyplex systems crucially affects their stability.

Conclusively this change in protonation, depending on the environment, affects both the stability and the efficacy of polyplex systems. Hence, controlling the effectiveness of a polyplex carrier system requires a delicate balance between stability and disassembly, immune evasion, and interaction with the endosomal membrane for endosomal escape.

The most common approach in the pharmaceutical sector to tackle this question is to formulate libraries and screen for biological performance. Yet, even when supporting such matrix screens with modern experimental design approaches or machine learning strategies, the wet lab effort remains high considering equipment, consumables, and personnel. *Vice versa* physicists, polymer scientists, or other fundamental researchers try to understand complex formation on a molecular level by *in silico* simulations such as molecular dynamics (MD) simulations. Despite the advantages of MD simulations, their application in the field of drug formulation is still in its infancy. Limitations include the vast computational resources required to simulate the complex systems at the relevant length and time scales and the need for accurate force field parametrization to ensure reliable results when using coarse graining approaches.^37,38^ Additionally, the correlation between simulated and experimental results often remains unaddressed.

Here, we present various approaches aimed at bridging the gap between fundamental molecular research and applied wet-lab techniques for investigating the thermodynamics of RNA-loaded polyplexes. We showcase a coarse-grained model for polyethylenimine (PEI) based on the Martini 3 force field. A systematic methodology is employed for the parametrization of PEI, which is essential for generating molecular dynamics (MD) files with PEI molecules across various molecular weights (MWs) and degrees of branching (DBs). Following an in-depth validation of our parametrization, we conducted complexation studies of different PEI homologues with small interfering RNA. These studies were approached using both MD simulations and a suite of complementary experimental methods, which included acid−base titrations, microscale isothermal calorimetry (micro-ITC) measurements, as well as fluorometric assays.

## 2 Materials and Methods

### 2.1 Chemicals

Polyethylenimines with molecular weight (*M*_W_) of 25 kDa (degree of branching (DB) = 59.0%), 5 kDa (DB = 58.4%), and 1.3 kDa (DB = 61.5%) were purchased from BASF (Ludwigshafen, Germany). Polyethylenimine with a *M*_W_ of 10 kDa (DB = 50.0%) was obtained from Polysciences Europe GmbH (Hirschberg an der Bergstrasse, Germany). SYBR Gold dye and black FluoroNunc 96-well plates were obtained from Thermo Fischer Scientific (Dreieich, Germany). Double-stranded siRNA targeting the enhanced green fluorescent protein gene (siGFP) was purchased from Integrated DNA Technologies (Munich, Germany). All other chemicals were obtained from Sigma-Aldrich (Schnelldorf, Germany).

### 2.2 Molecular Dynamics (MD) Simulations

The MD simulations of the parametrization part consisted of two simulation runs. The first is an all-atom (AA) run, in which the respective AA model, along with the TIP-3 water model, is simulated. For the first part, the system was minimized using the steepest descent algorithm for 500 steps and the tolerance for the energy minimization procedure of 100 kJ mol^−1^ nm^−1^. The systems were equilibrated with a time step of 10 fs and a total number of 250,000 steps using a leapfrog algorithm. Temperature was controlled by the Berendsen thermostat at a temperature of 298.15 K. Pressure was handled by the Berendsen Barostat with an isotropic pressure coupling. In the production runs designated as AA runs, a time step of 2 fs was employed, leading to simulations spanning 75,000,000 steps or a total duration of 150 ns. Coulombic interactions were addressed using a reaction-field method with a cutoff distance set at 1.4 nm. Van der Waals interactions were managed using a cutoff algorithm. Temperature regulation was achieved through a Nose-Hoover thermostat, utilizing a temperature coupling constant of 1.0 and the temperature at 298 K. Pressure maintenance was conducted via a Parrinello–Rahman barostat, set to sustain a pressure of 1 bar. All simulations were conducted using GROMACS 2022 series.^39,40^

Simulation parameters for the coarse-grained (CG) calculations were adapted from the established Martini Tutorial guidelines. Initially, energy minimization was performed utilizing the steepest descent method for a total of 2000 steps. Subsequently, an NPT equilibration was carried out for 25,000 steps, employing a time step of 20 fs. The Leap-Frog algorithm was utilized for the integration of the equations of motion during the equilibration phase.^41,42^ The Verlet neighbor search algorithm is used to update the neighbor list every 20 steps with a buffer tolerance of 0.005 kJ mol^−1^ ps^−1.43^ For the Lennard-Jones terms eq 3, we used a cutoff scheme with a value of 1.1 nm and the Verlet cutoff scheme for the potential-shift.^44^ Long-range electrostatic interactions were treated with a reaction field with the relative permittivity set to *ε*_r_ = 15 and a cutoff value of 1.1 nm. Periodic boundary conditions were used in all three dimensions. For the production simulations, the velocity rescaling thermostat (coupling time constant of 1.0 ps) and the Parrinello−Rahman barostat (coupling time constant of 12.0 ps) were employed to maintain temperature and pressure, respectively.^45,46^ Caused by the generation of complex hyperbranched structures, it could not be ruled out that beads are overlapping. Therefore, the first energy minimization in vacuum was performed using soft potentials which avoids the risk of calculated forces for the bonded interactions to become infinite.^47^ If not stated otherwise, a time step of 20 fs was used. Analysis was performed using *gmx* analysis tools (GROMACS 2022.3).

### 2.3 Titratable Martini

For the titration simulations, the polymer generator was adapted to meet the input requirements for the titratable Martini setup. The systems were prepared using Python scripts provided by Grünewald et al.^48^ All simulations were performed using the base titration setup. The size of the simulation boxes varied depending on the polymer size, with edge lengths of 5, 10, 12, and 16 nm used for polymer sizes of 1.3, 5, 10, and 25 kDa, respectively. Energy minimization was executed utilizing the steepest descent algorithm over a course of 800,000 steps. For the equilibration phase, the steepest descent (SD) integrator was employed, and the simulation was conducted for a total of 5,000,000 steps. In consideration of numerical stability, a reduced time step of 7.5 fs was implemented for polymer sizes below 25 kDa, in contrast to the conventional 20 fs time step typically used in Martini 3 simulations. Additionally, the time step was further reduced to 5 fs for the 25 kDa run due to observed instabilities. Electrostatic interactions were calculated using the particle-mesh Ewald (PME) method^49^ with a cutoff of 1.1 nm. The temperature was set to 298.15 K and controlled using the velocity rescale (V-rescale) thermostat, while pressure was maintained at 1 bar using the Parrinello−Rahman barostat.^46^ Simulations were run for a total of 37.5 ns, corresponding to 5,000,000 steps. Analysis was conducted using the provided analysis script as well as a custom *R*_g_ analysis script.

### 2.4 Complexation Studies

To conduct the complexation simulations, a setup was prepared in which an siRNA molecule was placed at the center of the simulation box with a 20 nm edge length, and a restraining force of 1000 kJ/mol was applied. Next, six simulation systems were generated with varying numbers of PEI molecules (1, 2, 3, 6, 11, 15), corresponding to *N*/*P* values of 0.6, 1.2, 1.8, 3.6, 6.6, and 9.6, respectively. Electrostatic interactions were calculated using particle-mesh Ewald (PME) with a cutoff of 1.1 nm. The temperature was set to 298.15 K and controlled using the velocity rescaling thermostat, while pressure was maintained at 1 bar using the Berendsen barostat. Simulations were run for a total of 1 *μ*s, corresponding to 83,333,333 steps with a time step of 12 fs to ensure numerical stability. The complexation energy was then calculated using the METADYNAMICS PLUMED^50^ plugin in the following steps: an axis was constructed between the first and last atom of the siRNA molecule, and the Gibbs free energy was measured as a function of the distance between this axis and the center of mass of the PEI molecule. To incorporate a small step of adsorption (2,000,000 steps), the PEI molecule of interest was restrained while the other PEI molecules were allowed to adsorb to the siRNA. In the metadynamics (MetaD) simulations, different bias factors were set based on the pH and *N*/*P* ratios. For simulations at pH 5.5, the bias factors were 100 for *N*/*P* ratios of 0.6, 1.2, and 1.8; 75 for 3.6 and 6.6; and 25 for 9.6. For simulations at pH 7.4, the bias factors were 75 for *N*/*P* ratios of 0.6, 1.2, and 1.8; 50 for 3.6; and 25 for 6.6 and 9.6. The Gaussian deposition rate was consistently set to deposit one hill every 100 steps, with a Gaussian width of 0.05 nm. These parameters were chosen based on best practices from a Martini 3 Meta D tutorial. Additionally, the appropriate bias factor was determined through trial and error to accurately represent both the association and dissociation behaviors of PEI in relation to siRNA. The sigma value was ascertained by conducting an unbiased simulation at an *N*/*P* ratio of 0.6 and calculating the standard deviation (0.0583 nm), a method consistent with the approach in previous studies performed on PEI siRNA interaction.^51^ Error analysis was conducted using the block analysis method detailed in the Plumed tutorials.^52^ Across all simulations, we observed a consistent pattern where the error margin expands until it stabilizes, reaching a plateau, beyond which it fluctuates indicating that the simulations have converged (see the Supporting Information (SI)).

In the next phase of our analysis, we integrated the potential of mean force (PMF) data from our simulations, correlating the results with corresponding *N*/*P* ratios. This integration process quantifies the complete range of energetic fluctuations inherent to the bPEI binding event, thus offering a detailed characterization of the interaction dynamics.

### 2.5 Acid−Base Titrations

First, solutions with the four different PEI samples were prepared to a concentration of 10 mg/mL in 150 mM NaCl. Next, 50 mL of the respective PEI solution was titrated with 1 N HCl in 2.5 mL steps under constant stirring. The pH was measured during the titration with a pH meter (Accumet AB 150, Fischer Scientific GmbH, Schwerte, Germany). Titrations were performed in triplicates.

### 2.6 Microscale Isothermal Calorimetry (Micro-ITC) Measurements

ITC measurements were performed on a Malvern MicroCal PEAQ-ITC device (Malvern Panalytical GmbH, Kassel, Germany). The concentrations of titrant (PEI) and cell sample (siGFP) were derived from previously published data on the titration of siGFP with PEI (0.05 mM base-pairs of siRNA, 3.0 mM nitrogen of PEI).^53^ However, the concentration of nitrogen in PEI was reduced to 1.0 mM to achieve curves that more closely covered the range around saturation of the binding event in this setup. Both reactants were prepared in 10 mM HEPES buffer with pH 5.5 and 7.4, which was sterile filtered with a 0.22 *μ*m syringe filter before sample preparation. Titrations were conducted at 298 K with a 19-step titration scheme and a reference power of 10 *μ*cal s^−1^. The volume for the initial injection was 0.4 *μ*L, followed by 18 injections of 2.0 *μ*L of PEI solution. The initial delay and time between injections was set to 150 s. Results were analyzed with the Malvern MicroCal PEAQ-ITC Analysis Software, using the One Set of Sites fitting model. Each fit was done regarding composite controls, including means of buffer-to-buffer, ligand-to-buffer, and buffer-to-cell material controls. Thermodynamic properties (N sites, dissociation constant *K*_D_, enthalpy Δ*H*, free Gibbs energy Δ*G*, and entropy −*T*Δ*S*) were calculated by the software from the curve fitted to the Integrated Heat Plot. Similar to previously published results,^53^ in some cases, endothermal enthalpies were observed in the injections close to saturation. To allow fitting of the curves, these injections had to be excluded from the analysis.

### 2.7 Polyplex Formulation and SYBR Gold Assay

To form polyplexes, first, siRNA and polymer were solubilized separately in 10 mM HEPES buffer (pH 5.5 or pH 7.4). The concentration of siGFP was fixed at 500 pmol mL^−1^, whereas the concentration of PEI was adjusted according to the desired *N*/*P* ratio. Both solutions were sterile-filtered with a 0.22 *μ*m syringe filter before being mixed in a volume ratio of 1:1 to allow for polyplex formation. For the SYBR Gold assay, 10 *μ*L of the respective polyplex formulations were transferred into a black FluoroNunc 96-well plate. Free siRNA (*c* = 250 pmol mL^−1^) was used as a 100% reference. Subsequently, 30 *μ*L of the SYBR Gold solution was added to each sample and incubated for 10 min. Fluorescence was measured with a plate reader (TECAN Spark, TECAN, Maennedorf, Switzerland) at 485 and 520 nm excitation and emission wavelengths, respectively.

### 2.8 Molecular Balance

For the single-molecule experiments, 1× phosphate-buffered saline (PBS) pH 7.4 with 420 mM NaCl and a 200 mM MES buffer pH 5.5 with 577 mM NaCl were filtered before measurements and addition of 2 mM Trolox and 20 mM EDTA. The buffer was oxidized with UV light to create Troloxquinone. Afterward, 2.4 mM PCA and the desired salt concentration were added, and pH was adjusted. For imaging, a 50× stock of PCD buffer containing 2.8 mM PCD (protocatechuate 3,4-dioxygenase from pseudomonas sp.), 50% glycerol, 50 mM KCl, 100 mM Tris HCl, and 1 mM EDTA-Na_2_·2H_2_O was mixed in a 1:50 ratio. The surface of a coverslip was cleaned with Hellmanex and passivated with BSA-biotin and neutravidin. Afterward, DNA origami with a biotin linker were immobilized on the surface, which was then washed twice with the above-mentioned buffer and once with the imaging buffer containing PEI in the desired concentration.^54^ The measurements were conducted using a total internal reflection fluorescence (TIRF) microscope (Olympus IX71, measurement time 60 s; exposure time 50 ms) as described previously.^55^ The DNA origami were folded and purified as described before.^56^

### 2.9 Representation of the Article

Data are visualized using Prism (version 9.5, GraphPad Software, Inc., Boston, MA), Affinity Designer 2 (version 2.0, Serif Ltd., West Bridford, U.K.), PyMOL (version 2.5, Schrodinger, Inc., New York, NY), and Blender (version 3.4.1, Blender Foundation, Amsterdam, The Netherlands) using the MolecularNode plugin (version 2.10.0). Some figures comprise graphical items created with BioRender. ChatGPT was used to suggest improvements of some of the text passages.

## 3 Results and Discussion

To model polyethylenimine (PEI) and small interfering (si)RNA, we chose the Martini force field which had recently been updated to its third version.^57^ In brief, the coarse graining (CG) process via the Martini model requires mapping of 2 to 5 heavy atoms of the all-atom (AA) structure into a single bead. These beads interact via both bonded and nonbonded interactions.^57,58^ To model PEI, harmonic bonded and cosine angle potentials were used which are given by

(1) $$V_{\text{bond}}(R)=\frac{1}{2}K_{\text{bond}}{\left(R-R_{\text{bond}}\right)}^{2}$$

and

(2) $$V_{\text{angle}}(\theta )=\frac{1}{2}K_{\text{angle}}{\left\{\text{cos}\;\theta -\text{cos}\;\theta_{0}\right\}}^{2}$$

where *K*_bond_ [kJ mol^−1^ nm^−2^] and *K*_angle_ [kJ mol^−1^] denote the force constants for bonds and angles, respectively. Thereby, *R*_bond_ [nm] and *θ*_0_ [deg] describe the equilibrium bond and angle position. Mapping of the siRNA additionally required the parametrization of improper and proper dihedrals, as well as constraints.

Nonbonded interactions are described by a Lennard-Jones (LJ) 12−6 potential (*V*_LJ_) and a Coulombic potential (*V*_Coul_), the latter of which only contributes to charged moieties eq 3.

(3) $$V_{\text{LJ}}\left(r_{1}\ldots r_{n}\right)+V_{\text{Coul}}\left(r_{1}\ldots r_{n}\right)=\underset{i,j}{\sum }4\varepsilon_{ij}\left[{\left(\frac{\sigma_{ij}}{r_{ij}}\right)}^{12}-{\left(\frac{\sigma_{ij}}{r_{ij}}\right)}^{6}\right]+\underset{i,j}{\sum }\frac{q_{i}q_{j}}{4\pi \epsilon_{0}\epsilon_{\text{r}}r_{ij}}$$

where *ε* [J mol^−1^] describes the depth of the trough of the LJ potential and *σ* [nm] is the distance at which the potential energy between two beads becomes zero. Within the Coulombic term, *q* describes the bead charges and *ϵ*_0_ and *ϵ*_r_ [F m^−1^] describe the permittivities of vacuum and solvent, respectively.

### 3.1 Coarse Graining Branched Polyethylenimine

To map branched polyamines such as PEI, a common approach is to differentiate beads that represent primary, secondary, and tertiary amines. Due to the limitations of the previous Martini 2 force field, however, PEIs were problematic to model and it was shown that a mapping of N−C−C units instead of the more intuitive C−N−C monomers has distinct advantages for the simulations.^59^ When compared to the previous Martini version, the update comprises several new bead types and subtypes. Especially the bead size, i.e., regular, small, and tiny is better differentiated allowing more detailed mapping of complex structures.

**Figure 1:**
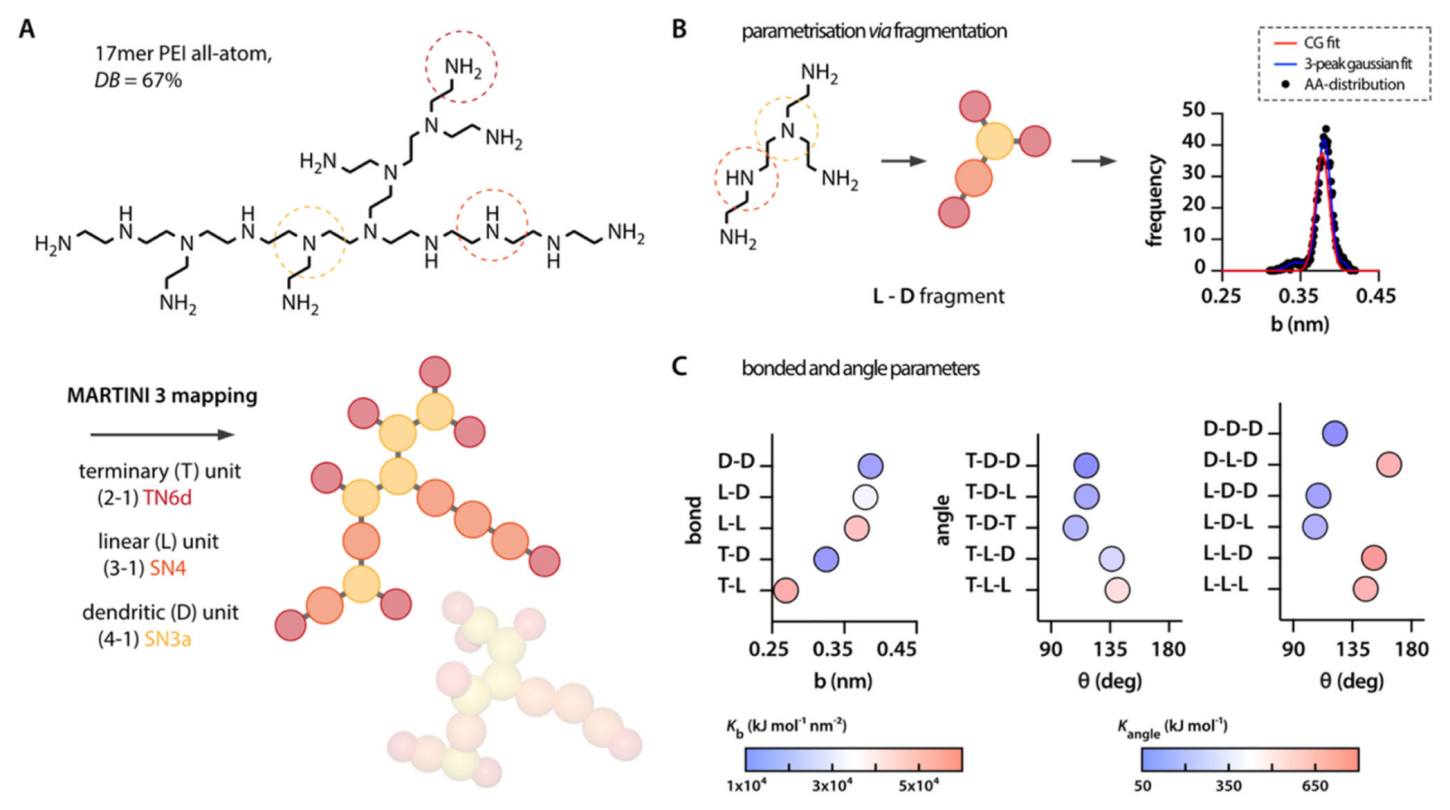
PEI mapping and parametrization. (A) A PEI molecule comprising all combinatorically possible variations in bond and angle arrangements comprises 17 differently arranged monomers. The molecule is mapped following the Martini 3 rules using three different bead types. (B) Fragmentation of the molecule and analysis of the corresponding AA-distributions allows for (C) extracting the bonded parameters for each of the bond and angle combinations.

This enabled us to adapt a direct mapping of the chemical monomers: although we used a tiny N6d type for terminal primary amines, we employed small beads of the types N4 and N3a for secondary and tertiary amines, respectively. The protonation of PEI at different pH levels, primary amines, and secondary amines is represented by charged beads, i.e., TQ5d and SQ5d, respectively (Table 1). As we here intended to simulate PEI molecules in their actual size of several kDa, this manual work is not sustainable. To be able to systematically generate MD files for larger PEI molecules we had to identify a more general approach to map the molecules. Therefore, we established a fragmentation strategy based on representative PEI models. This representative PEI model was designed to comprise all possible bond and angle combinations with linear (L) dendritic (D) and terminal units (T). The PEI resulting from this combinatorics exercise comprises 17 monomers with a degree of branching (DB) following the definition of Hölter et al.^60^

(4) $$\text{DB}=\frac{2\cdot \text{D}}{2\cdot \text{D}+\text{L}}\text{or}\frac{\text{DB}}{\left(1-\text{DB}\right)}=\frac{\text{D}}{\text{L}}$$

of 67% (Figure 1A, “bPEI67”). According to this mapping scheme, we then generated parametrization for both coarse-grained and the corresponding AA fragments considering bond (5), e.g., L-D, and angle (11), e.g., T-L-D. Open ends of those fragments were capped with terminal units (Figure 1B).

In the first step of the parametrization, AA simulations were conducted to extract bonded and angle distributions for each of the 17 5-mer fragments (Figure 1B, black dots). For this purpose, we implemented a strategy that has previously been shown to be suitable for defining bonded interactions in a systematic manner;^61^ in brief, each frequency distribution was fitted with a 3-peak Gaussian distribution (example in Figure 1B, blue curve; complete data set in the SI)

(5) $$P=\sum_{i=1}^{3}A_{i}{\text{e}}^{-V_{i}//k_{\text{B}}T}$$

where *P* represents the probability distribution, *V*_*i*_ is the potential energy distribution for either bond (eq 1) or angle (eq 2), *k*_B_ is the Boltzmann constant (in Gromacs *k*_B_ is used identically to the gas constant *R* = 8.314 J mol^−1^ K^−1^), and *T* is the absolute temperature [K].

As the Martini model does not allow representing distributions with multiple peaks, a reasonable transformation needed to be conducted and we used a weighted average function from the 3-peak fits (Figure 1B, red curve; complete data set in the SI) according to

(6) $$w_{i}=\frac{S_{i}}{\Sigma_{i}S_{i}}$$

where *w*_*i*_ represents the weight of the individual peaks proportional to the peak area under the curve *S*_*i*._ This single-peak Gaussian function was then used to extract the equilibrium bond and angle values as well as their corresponding force constants (Figure 1C) following:

(7) $$\begin{array}{c} \frac{1}{K_{\text{bond}}}=\sum_{i=1}^{3}\frac{w_{i}}{K_{\text{bond},i}},b_{0}=\sum_{i=1}^{3}w_{i}b_{0,i}\;\text{and}\;\frac{1}{K_{\text{angle}}}\\ =\sum_{i=1}^{3}\frac{w_{i}}{K_{\text{angle},i}},\theta_{0}=\sum_{i=1}^{3}w_{i}\theta_{0,i} \end{array}$$

Values for *K*_*i*_ < 1 and *w*_*i*_ < 0.1% were excluded as they tended to affect the reduced parameters overly strongly despite that they did not play a significant role in the quality of the fit. Notably, the parametrization was conducted in the absence of any charged groups. As we intend to use that parametrization for generating random structures, this was necessary to avoid overlaying Coulombic forces from charged groups. Such overlaying forces would shift the recorded bond angle distributions and thus make it impossible to extract a general parameter set for the different bead combinations. As the Martini model simply superposes bonded and nonbonded interactions (eq 4), the addition of charges at a later point will automatically reflect in the resulting distributions.

### 3.2 Validating the Parametrization

**Figure 2:**
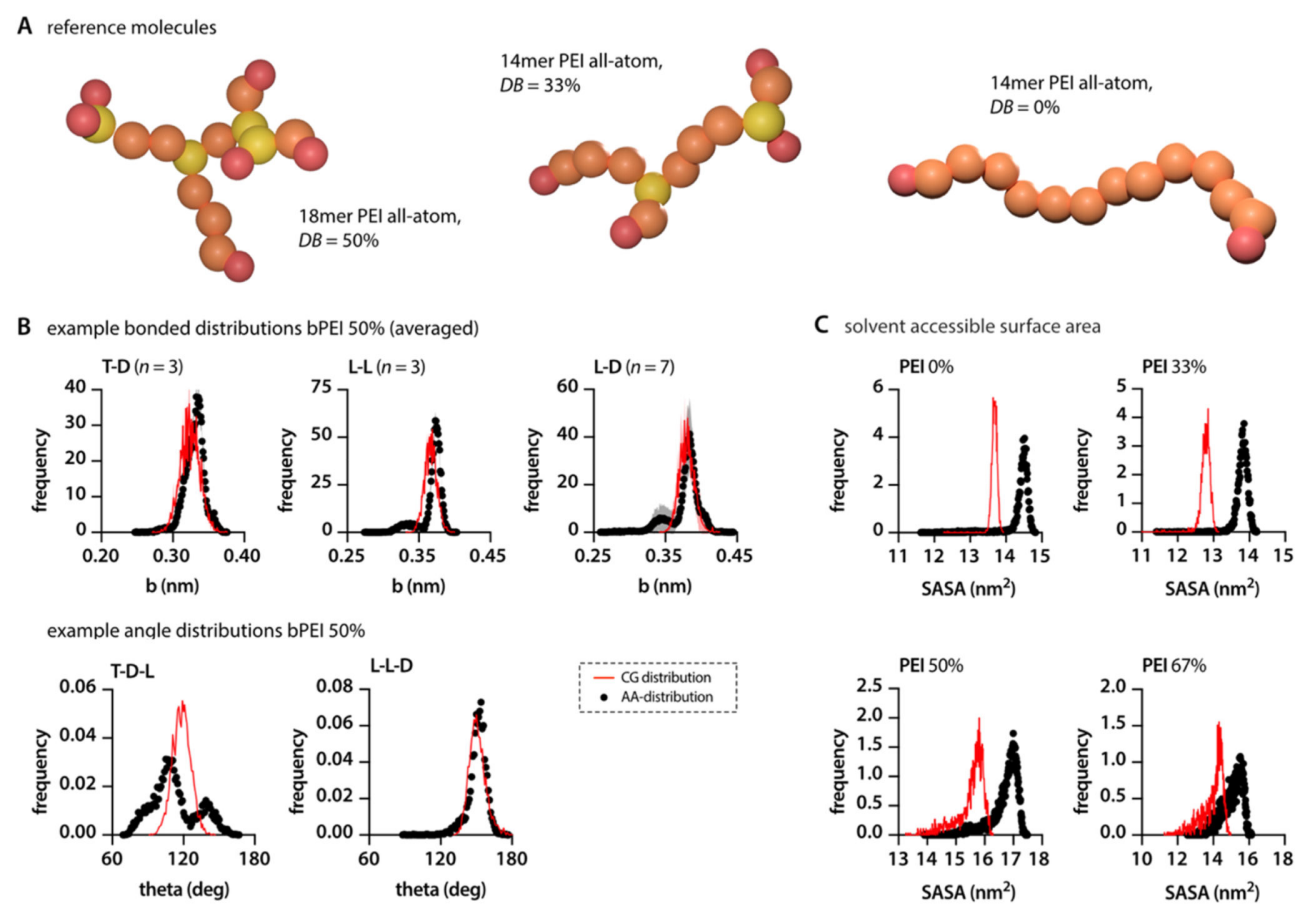
Validation of the PEI model. (A) Additional PEI structures that can be differentiated regarding their DB were designed to validate the parametrization. (B) Comparing the bonded and angle distributions of AA and CG simulations for bPEI50 confirms the accuracy of the parametrization. Example bonds presented here (T-D, L-L, L-D) represent mean ± SD from all similar bonds within the test molecule. (C) Solvent-accessible surface area in CG simulations is slightly lower when compared to the corresponding AA runs.

In the second step of our parametrization, the bond and angle distributions of the 17 5-mers were used as a benchmark to compare against the coarse-grained (CG) simulations of the larger 17-mer model molecule bPEI67. To further substantiate our findings and test the performance of the parametrization, we extended this comparison to three additional model molecules, each with a different degree of branching (DB): 50% (bPEI50), 33% (bPEI33), and 0% (PEI0) (Figure 2A).

Comparing the bond and angle distribution of the CG simulations with those obtained from the AA runs, we observed an impressively high agreement (see Figure 2B for examples, and the SI for complete data set). Only in a few cases, especially when terminal units were present, some deviations of the extracted CG-distributions from the AA results were observed. The deviations are expected to originate from a higher sensitivity toward the influence of neighboring groups rather than from an inaccurate parametrization. Since the aim of this study was to simulate PEIs in their common molecular size of 1−25 kDa, we expect those divergences to statistically level themselves out. Furthermore, as expected, multimodal distributions, which are often more pronounced in angle distributions, were represented by a single peak in the weighed center of the different peaks (Figure 2B). Overall, the parametrization was similarly accurate, independent of the DB of the PEI.

We additionally assessed the solvent-accessible surface area as a validation parameter for the CG model. For all four PEI homologues simulated here, the CG model slightly under-represented the solvent-accessible surface area by 5.0 to 7.5% (Figure 2C). In agreement with previous studies using the Martini force field^62^ and the tutorials published on the developers’ webpage,^63^ these deviations can be considered acceptable.

### 3.3 Polymer Generator, Charge Integration, Titration

**Figure 3:**
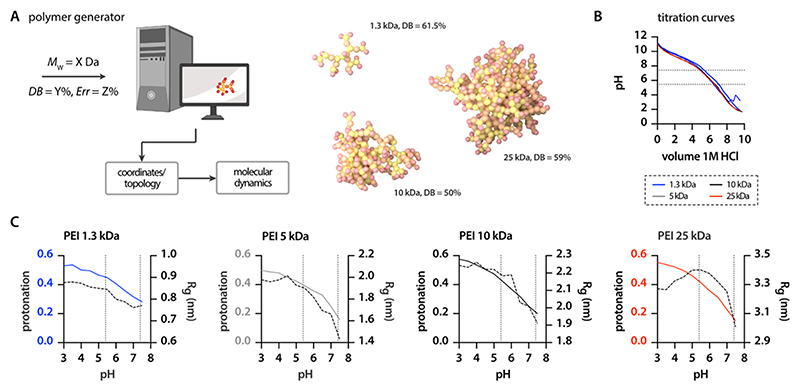
Simulation-file generator and titration models for PEIs. (A) Using very few user inputs the polymer generator can produce all files that are necessary to run CG simulations of large, kDa-sized PEI molecules. (B) Titration curves of four commercially available PEIs with different *M*_W_’s and BDs are virtually identical without indicating sharp turning points/p*K*_a_ values. (C) Titration simulations depict comparable trends for the pH-dependent protonation (colored lines) of the different PEIs and show the influence on their conformation (dotted lines).

The generation of both linear polymer and perfect dendrimer structures for MD simulations is rather straightforward and can be performed manually or automatically via different tools such as the commercial suite Schrodinger Materials Science and different open-source codes.^64,65^ Although different studies previously generated hyperbranched polymer models in an automatized manner,^66^ the adaptability for specific applications, in our case PEI, is limited and time-consuming. Consequently, we developed a script (TheGenerator.py, brief description and download link in the SI) to automatically create input files for branched PEI molecules with adaptable DB and molecular weight (*M*_W_) based on the above explained mapping scheme. Starting from an initiator bead—a secondary amine with two open ends—our script allows the polymer structure to grow consecutively on all branches, thus mimicking the chemical polymerization process.^67,68^ Polymer growth continues until the target *M*_W_ is reached. Whether a dendritic node (D, tertiary amine) or a linear node (L, secondary amine) is added to the branch is controlled by a probability function derived from the user-defined DB (eq 5). Furthermore, the user defines a termination probability, i.e., the probability that a branch is terminated with a primary amine instead of being continued (Figure 3A). As, depending on this probability, a premature end of the polymerization process cannot be excluded multiple attempts (default = 100) are performed to increase the probability that the target weight is reached. The program generates a coordinate file and a topology file according to the GROMACS terminology. Furthermore, mapping files are generated as well as a smiles string to be able to reconstruct the corresponding AA files.

As described in Section 3.1, we initially neglected the role of charges in the conformation and physicochemical properties during the parametrization of the PEI molecules, as the superposition of bonded and nonbonded interactions in the Martini model (eq 4) allows for investigating the influence of charged groups independently from the parametrization of bonded interactions. Concerning the influence of charge on bonded distributions, Gallops et al. have highlighted that increased protonation can extend the N−N distance at elevated protonation levels and lead to modifications in the N−C−C−N dihedral angles, primarily resulting in a trans conformation.^69^ However, in our study, the use of a coarse-grained force field limits our ability to depict such detailed conformational shifts. Furthermore, these changes predominantly occur at very high protonation percentages, where adjacent amines are protonated. Our focus, though, is on pH levels of 5.5 and 7.4, which do not correspond to these high protonation levels and the formation of doublets.

Nevertheless, due to the different types of amines and their varying p*K*_a_ values, PEI is known to exhibit a strongly charge-dependent behavior. This phenomenon was also investigated in the work of Ziebarth et al.^70^ To better represent and comprehend the protonation process, we adapted our CG models following the Titratable Martini method recently developed by Grünewald et al.^48^ Although the Titratable Martini model is still emerging, it has successfully depicted the protonation-structure relationship in charged dendrimers such as poly(propyleneimine). The titration model introduces a reduced number of beads, namely, “titratable beads”, which change their charge after surpassing a predefined p*K*_a_-value. Selecting the correct p*K*_a_-value for the respective groups, however, is not straightforward. It was often assumed that mainly the primary amines of PEI are protonated followed by the secondary ones when the pH is lowered.^71^ With aliphatic substituents, however, the p*K*_a_ value is expected to increase in the order p*K*_a_ (primary) < p*K*_a_ (secondary) < p*K*_a_ (tertiary), which is likely to be due to the inductive effect; the more aliphatic chains are substituted with nitrogen, the higher their electron density resulting in stronger proton-acceptance.^72^ This in contrast would indicate that tertiary amines become protonated first. This contradiction might originate in the proximity of the amine groups within the PEI structure, which allows single amines to considerably affect the protonation of neighboring amines. Furthermore, steric shielding of the respective amine groups could convert the inductive effect.^73^

We performed experiments on four different commercially available types of PEI that vary regarding *M*_W_ and DB (see Section 2). Overall, the obtained titration curves look similar, independent of the PEI MW. The slope of the curves seems to slightly change twice, between pH 7 and 8 and at around pH 5. The curves further indicate the highest buffering capacity to be in the neutral region. The absence of sharp turning points supports our previous discussion that p*K*_a_-values cannot be clearly assigned to the different amine types but vary broadly due to the interaction with neighboring structures (Figure 3B).

To better understand the complex protonation behavior, we used density functional theory (DFT) to predict the p*K*_a_-values of selected amines. DFT analysis confirmed our expectation that steric shielding hampers protonation. For example, a tertiary amine located at the distal end of a PEI model had a p*K*_a_-value of 9.1, whereas a group located more in the center of the molecule showed a p*K*_a_-value of 8.6 (see the SI). As a complementary approach, we utilized the EpiK module in the Schrodinger software Bioluminate to determine the p*K*_a_-values and protonation states. Our analysis revealed p*K*_a_-values of 7.0 and 7.1 for the tertiary amines in the 14-mer polymer bPEI33. For the four primary amines, EpiK calculated p*K*_a_-values between 9.3 and 9.4. The p*K*_a_-values of the secondary amines showed a broad distribution ranging from 5.3 to 9.0.

We tried to reflect the protonation behavior reported by different references^70,74−76^ and simulated these protonation states using *in silico* tools in our titratable model. Limited by the boundary condition that the titratable model comes with a strongly reduced number of different beads, we chose titratable beads with a p*K*_a_-value of 10.6 for primary amines and 10.2 for secondary and tertiary amines. After generating CG files of polymers with MWs of 1.3, 5, 10, and 25 kDa using the adapted version of “The Generator”, titration simulations were performed in the accessible pH range of 3 to 8. For all PEI molecules, the degree of protonation changes from 40 to 60% at acidic pH to a minimum of around 10% at neutral pH. Below endosomal pH, i.e., <5.5, the degree of protonation is only slightly affected by pH. In contrast, drastic changes occur between pH 5.5 and 7.4. Driven by electrostatic repulsion, the radius of gyration (*R*_g_) of the branched PEI structures increases by 20 to 30% depending on the *M*_W_ and DB of the PEI when lowering the pH (Figure 3C).

### 3.4 Complexation of Small Interfering RNA with PEI

To perform complexation studies with our newly parametrized PEI model, we prepared a corresponding model of siRNA. Specifically, we aimed to model a GFP targeting siRNA sequence available for wet-lab techniques (see the SI for sequence and base modifications). As of now, no Martini 3 model for siRNA is available. Based on the Martini 2 RNA, the martini-nucleotide.py script^77^ was adapted to incorporate the new parameters for Martini 3 nucleobases. Currently, there are no available parameters for the backbone so that we manually converted the bead type to the corresponding Martini 3 bead type. Since the siRNA model comprises a multitude of different bead types, it was not possible to create this model within the limited framework of the titratable Martini force field. Therefore, a protonated PEI model also had to be implemented in the standard Martini 3 framework.

**Figure 4:**
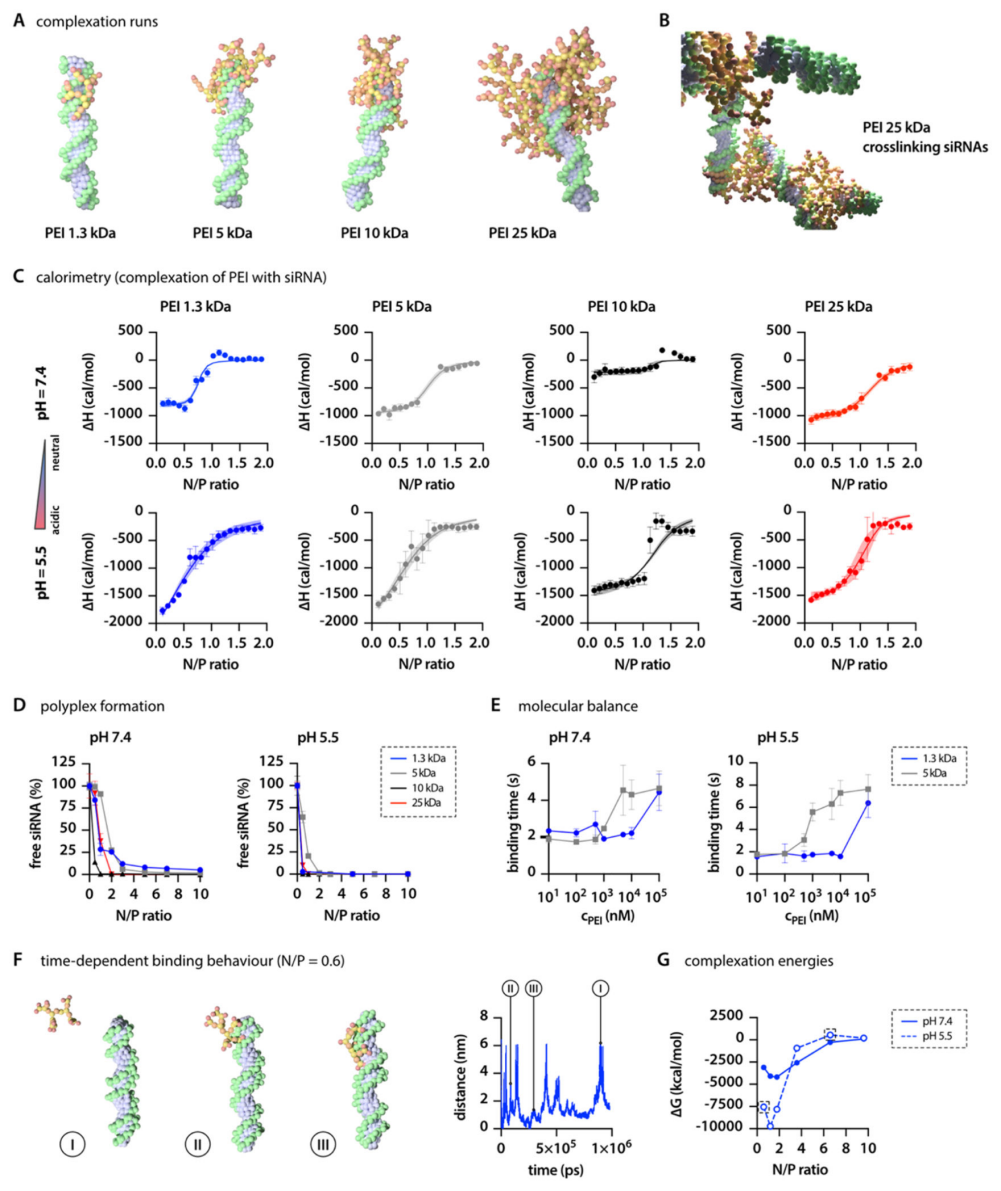
PEI−RNA interactions. (A) Depiction of siRNA complexation with various *M*_W_ PEIs, highlighting the distinct structural interactions. (B) Multiple siRNAs cross-linked via PEI bridges, revealing the capability of PEIs to connect several RNA molecules. (C) Complexation thermodynamics for different PEIs, as quantified by micro-ITC analysis (*n* = 4). (D) Examination of the relationship between *N*/*P* ratio and unbound siRNA in complexation behavior as determined by fluorescence intercalation, demonstrating the efficiency of complexation across various ratios (*n* = 3). (E) Investigation of the energy landscape of DNA/PEI complexation at two distinct pH levels measured using single-molecule spectroscopy of molecular balances. (F) Binding/unbinding events of 1.3 kDa PEI to siRNA over time. (G) Influence of pH on the complexation free energy Δ*G*, derived from MD simulations at increasing N/P ratios and two distinct pH levels.

For our studies, we decided to focus on two different scenarios, pH 7.4 representing the extracellular and cytosolic pH, and pH 5.5 reflecting the pH of the endolysosomal compartment. Accordingly, we added a feature to “The Generator” where the user can choose between these two preset pH levels. Choosing neutral pH first leads to the code replacing 66% of the primary and 33% secondary amines with their corresponding charged beads (see Table 1). Selecting the more acidic pH leads to protonation of 100% of primary, 66% of secondary, and 33% of tertiary amines. We observed distinct differences in complexation behavior for PEI of different sizes and depending on pH. Smaller PEI molecules, particularly those with 1.3 and 5 kDa, closely wrap around the siRNA molecule, while larger PEI homologues extend into the surrounding (Figure 4A). As a result, larger PEI molecules, such as the 10 kDa homologue, can form complexes with multiple RNA molecules (Figure 4B).

Thermodynamically, electrostatic complex formation is characterized by a reduction in the enthalpy *H* of the system and a decrease in the system’s entropy *S*. However, the Gibbs free energy Δ*G* = Δ*H* − *T*Δ*S* decreases overall, making electrostatic complexation a thermodynamically favorable state.^31^ Experimentally, this saturation behavior can be assessed using isothermal titration calorimetry. Standard binding isotherm curves depict enthalpically favorable binding that saturates at N/P ratios between 1 and 1.5. The absolute enthalpy change is considerably larger for pH 5.5 compared to pH 7.4, emphasizing the stronger binding interactions at endolysosomal conditions. However, marked energetic differences between different *M*_W_ PEIs were not observed (Figure 4C). Data from the isotherms confirm the overall negative enthalpy values (Δ*H*) upon complexation, indicating stronger binding at acidic pH (Table 2). PEI protonation is known to increase with decreasing pH, while the RNA’s phosphate group deprotonation remains unaffected at the pH levels of interest. Thus, stronger Coulombic forces are expected at endosomal pH than at cytosolic pH. The point at which the complex achieves overall charge neutrality is a critical metric for all polyplex systems, reflecting thermodynamic equilibrium, with saturation anticipated at this point. Lower *N*/*P* ratios can achieve charge neutrality when pH is lower. Previous studies, including Brownian simulations, have demonstrated that a pH decrease can result in polymer release from the polyplex,^78^ as the amount of polymer required for saturation decreases. Experimental studies have also confirmed polymer shedding from polyplexes when the pH level is lowered.^79^ For drug delivery systems, the amount of polymer needed to saturate the complex is important as the endosomal escape of polyplexes is related to the release of polymer molecules, which disrupt the endosomal membrane.^80^

Notably, we observed negative entropy terms (−*T*Δ*S*) under all conditions, contradicting the thermodynamics of purely electrostatically driven complex formation (Table 2). This increase in entropy (Δ*S*) suggests a hydrophobic contribution to the assembly process. Additionally, the loss of hydration and counterions in contact with both polyelectrolytes may further increase entropy in the system and contribute to the overall favorable complexation. These effects strongly depend on the ionic strength of the buffer system.^81^ Previous observations indicate that, on the atomic level, cation-RNA interactions are dominated by electrostatic forces, while hydrophobically driven assembly processes contribute to the overall increased stability of polycation-RNA complexes at larger scales.^53,82^ A similar pH-dependent effect of the complexation process can be observed in wet-lab experiments when complexing siGFP with PEI at different *N*/*P* ratios and determining the amount of free siRNA. Full complexation (>95% of RNA is bound) is achieved between *N*/*P* = 1 and 5 at neutral pH and 0.5 and 2 at endolysosomal pH (Figure 4D).

To further investigate the binding behavior between PEI and nucleic acid molecules, we employed an exploratory approach utilizing single-molecule fluorescence spectroscopy on a molecular balance. This molecular balance consists of a DNA origami structure^54^ with a protruding pointer strand that can transiently bind to two additional protruding strands (both ssDNA).^56^ The kinetics of the molecular balance are monitored *via* fluorescence resonance energy transfer (FRET) between a Cy3B donor dye attached to the end of the pointer strand and an ATTO647N acceptor dye adjacent to one binding site. This setup enables the observation of transient hybridization kinetics between low- and high-FRET states with high-FRET contrast. As the molecular balance operates at thermal equilibrium, the kinetics are highly sensitive to the energy landscape of the hybridization; even a small alteration in the sequence of the binding site can change the kinetics by more than an order of magnitude.^56^

In the presence of PEI, the hybridization of pointer and binding site is stabilized, thus increasing the binding times which represents a decrease of the overall Gibbs free energy. As the PEI concentration increases, and consequently the *N*/*P* ratio, a saturation behavior of the hybridization times is observed. With increasing PEI MW from 1.3 to 5 kDa, the onset of this saturation behavior shifted to lower concentrations. Although hardly any pH differences can be observed for the 1.3 kDa PEI homologue, we identified that decreasing the pH and thus increasing the protonation shifts the saturation to lower concentrations for larger PEI molecules (Figure 4E). Thus, the molecular balance results qualitatively agree well with the ITC and the free siRNA intercalation analyses results. It is important to note that the molecular balance was used to investigate the complexation of PEI with DNA rather than RNA. Considering the electrostatic and hydrophobic interactions as the two primary driving forces for the complexation of nucleic acids with PEI, and given the molecular similarity of RNA and DNA, we do not expect substantial differences in the global energy readouts. However, it is important to note that geometric considerations, such as the accessibility of charged groups, can differ among nucleic acid-based molecules. These differences can indeed influence their complexation behavior with polycations.^33^

Metadynamics simulations at the AA level have proven to be potent in precisely assessing the thermodynamics of binding between polycations, such as PEI, and siRNA.^51^ Indeed, such detailed analysis can serve as a robust tool for a deeper understanding of thermodynamic data acquired experimentally. Conversely, deriving quantitative values from complexation studies at the CG level that can be directly correlated with thermodynamic values from ITC can be challenging. We propose a methodology to assess binding interactions *in situ*. We performed simulations that mimic measurements to determine binding forces at a single-molecule level. As described in Section 2, we fixed the position of a single siRNA molecule in a simulation box and allowed PEI molecules (at varying *N*/*P* ratios) to complex with this siRNA while one bPEI molecule was restrained at a 6 nm distance (COM-DOA). After the complexation process reached equilibrium, we lifted the restrains of the observed bPEI molecule and let it absorb and desorb using Metadynamics. Upon examining the fluctuation between the bound and unbound states of individual PEI molecules (Figure 4F), the simulations reveal that higher *N*/*P* ratios result in increased repulsion. This, in turn, makes the binding of a specific bPEI molecule less favorable (see the SI). Therefore, the energy required to retract a single PEI molecule (see the SI for the individual potential of mean force (PMF) curves) decreases with increasing *N*/*P* ratio as the complex becomes saturated with polymer (Figure 4G). Additionally, we observed considerable differences in the absolute energy values of the complexation when decreasing the pH to 5.5 (Figure 4F). While there are noticeable differences in the absolute values of Δ*H* and Δ*G* derived from isothermal titration calorimetry (ITC) measurements compared to those obtained computationally, the congruence in their sigmoidal nature and distinct behavior at two separate pH levels offers important insights. Additionally, the experimental data of the Molecular Balance experiments indicates longer binding duration at higher *N*/*P* ratios, a trend that aligns with the energetically less favorable outcomes at similar ratios observed in Metadynamics simulations, as shown in the energy curves detailed in Supporting Figure 7. The significant discrepancies observed in the Δ*G* values between experimental results and simulations may originate from several factors. Notably, the Martini 3 force field sets the solute screening factor (epsilon_r) at 15, which contrasts with the actual dielectric constant of water, approximately 80. This discrepancy could artificially extend the range of electrostatic interactions. Additionally, capturing the full spectrum of energetic influences, such as hydration shell disruption and counterion dynamics around polyelectrolytes, would require a model with higher resolution of the model. Furthermore, it was not possible to transfer this methodology to larger PEI molecules, as it would be necessary to add additional siRNA molecules into the box to obtain the incremental *N*/*P* ratios, which would then interfere with the retraction process.

An additional aspect that remains to be considered is the role of flexibility and geometry in these complex molecular interactions. Previously, Grasso et al. demonstrated in all-atom (AA) simulations that polycations containing amide bonds exhibit reduced flexibility due to the rigidity imparted by these bonds. Conversely, branched polyethylenimine (bPEI) showcases greater angular flexibility, which enables it to envelop siRNA more closely, thereby facilitating stronger interactions.^51^ Studies investigating either the polymer component^83^ or the nucleic acid component^33^ further underscore the significance of polymer flexibility and molecular geometry in the complexation process with nucleic acids. While we derived angle parameters from AA simulations, our CG model lacks the resolution to fully capture every nuance of these interactions. However, the capability to generate true-sized PEIs with varied degrees of branching in our model allows for future exploration of how these higher-level geometric considerations impact the complexation process. This is a critical aspect that warrants further examination in subsequent studies.

## 4 Summary and Conclusions

The design of drug carrier systems often requires polymers to be tailored for specific uses, emphasizing the importance of understanding their structure−function relationships. Traditionally, this design process is based on creating large libraries and their empirical optimization, which is a time- and cost-intensive method. These drawbacks highlight the advantage of using computational tools for a more streamlined and precise drug carrier development.

In our study, we developed a CG model for PEI using the Martini 3 force field. We performed MD simulations to study the complexation between siRNA and PEI. Our goal was to combine computational and experimental methods to obtain a comprehensive view of the thermodynamics of RNA complexation with polycationic carriers.

Our findings show that the PEI CG model accurately represents the polymer properties. Simulations indicated that electrostatic interactions are central to PEI and siRNA complexation, and that molecular weight and branching of PEI play a pivotal role in this process. Notably, while electrostatic forces were dominant, entropic considerations also emerged as key contributing factors. By using both computational and experimental methods, our study points to the value of a combined approach in understanding polymeric drug carrier interactions. The results highlight the benefits of computational tools in the design process. Such tools offer a more efficient and precise method, suggesting a shift toward a modern, rational design strategy. Our research suggests a roadmap for creating specific polymeric drug carriers. This strategy, backed by solid *in silico* data, emphasizes the potential of computer-aided over traditional empirical and unstructured library approaches.

## Supplementary Material

Supplementary code

Supplementary information

## Figures and Tables

**Table 1:** Mapping of PEI^a^

chain element	group	atoms	charge	Martini 3 bead type
terminal unit (T)	primary amine	H_2_N−CH_2_	0+1	TN6d TQ5d
linear unit (L)	secondary amine	H_2_C−NH−CH_2_	0+1	SN4 SQ4p
dendritic unit (D)	tertiary amine	H_2_C−NCH_2_−CH_2_	0+1	SN3a SQ3p

a Atom to bead mapping of the different chain elements (characterized by their amine group) and corresponding Martini 3 bead types both in their charged and uncharged state. Their respective Lennard-Jones parameters can be found in the Martini 3 force field file on GitHub https://github.com/marrink-lab/martini-force_fields/tree/main/martini_forcefields/regular/v3.0.0/gmx_files.

**Table 2:** Calorimetry Details^a^

PEI size (kDa)	pH	N (sites)	*K*_D_ (*μ*M)	Δ*H* (kcal/mol)	Δ*G* (kcal/mol)	−*T*Δ*S* (kcal/mol)
1.3	7.4	1.41 ± 0.03	0.70 ± 0.13	−0.82 ± 0.02	−8.41 ± 0.11	−7.59 ± 0.01
	5.5	1.33 ± 0.27	32.4 ± 8.2	−2.65 ± 0.27	−6.29 ± 0.18	−3.64 ± 0.41
5.0	7.4	1.97 ± 0.10	2.94 ± 0.64	−0.97 ± 0.02	−7.57 ± 0.14	−6.60 ± 0.15
	5.5	1.34 ± 0.28	20.7 ± 8.1	−2.38 ± 0.37	−6.44 ± 0.23	−4.10 ± 0.60
10	7.4	2.20 ± 0.14	0.26 ± 0.14	−0.22 ± 0.06	−9.11 ± 0.49	−8.89 ± 0.54
	5.5	2.16 ± 0.09	1.15 ± 0.97	−1.39 ± 0.12	−8.42 ± 0.85	−7.03 ± 0.95
25	7.4	2.38 ± 0.10	5.00 ± 1.53	−1.09 ± 0.05	−7.26 ± 0.19	−6.17 ± 0.18
	5.5	2.01 ± 0.21	3.72 ± 2.29	−1.60 ± 0.14	−7.53 ± 0.48	−5.93 ± 0.62

a Calorimetric parameters (N-sites, *K*_D_, enthalpy change, Gibbs free energy change, entropy change) as obtained *via* micro-ITC for the complexation of siRNA with different PEIs at two pH levels. Data indicate mean ± SD (*n* = 4).
