# Supplementary figures and images for: Closing the Gap between Experiment and Simulation—A Holistic Study on the Complexation of Small Interfering RNAs with Polyethylenimine
Source: Mol Pharm. Author manuscript; Available in PMC 2024 Oct 29. (PMC7616749; doi:10.1021/acs.molpharmaceut.3c00747)

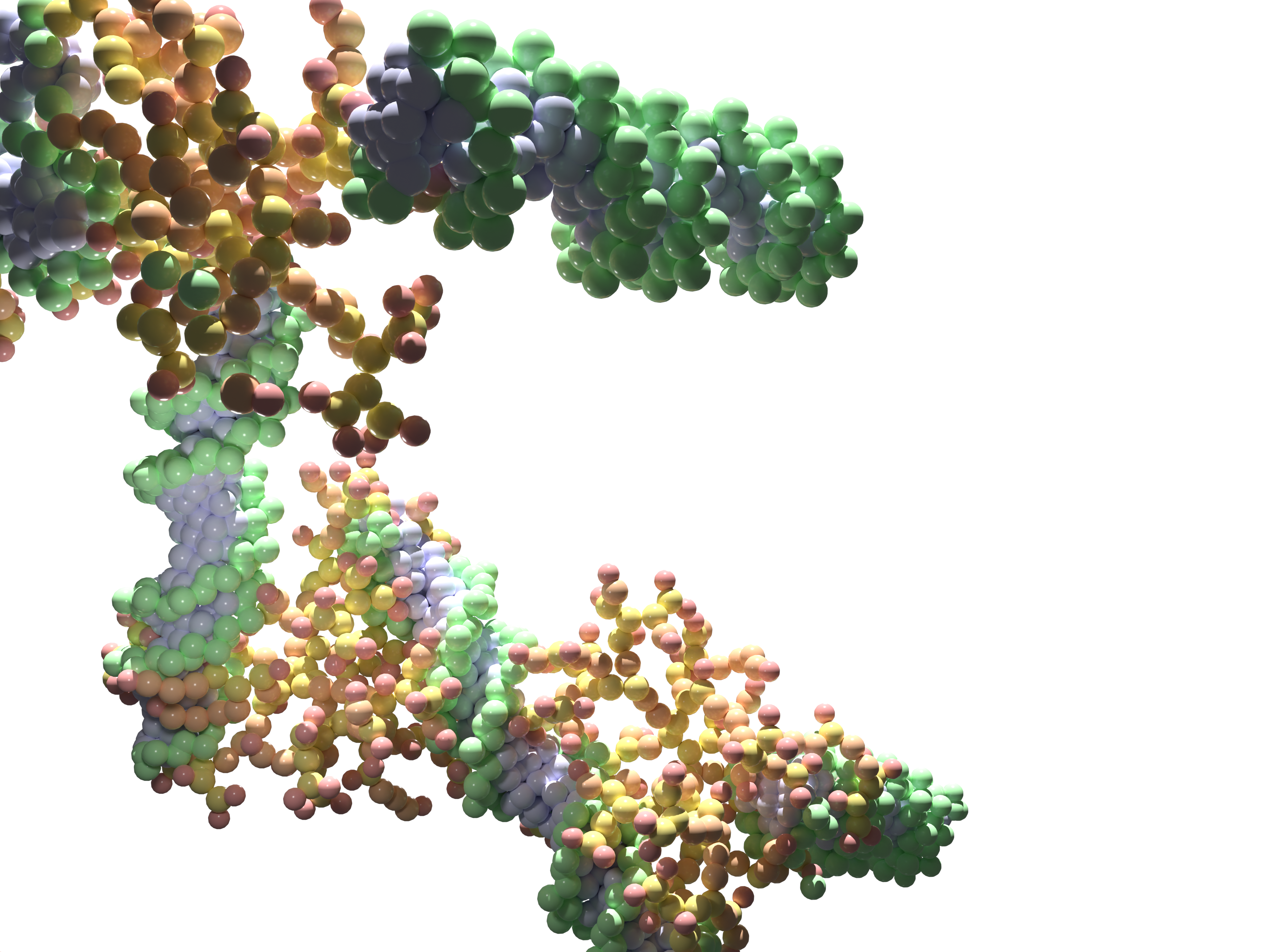

Supplement: Supplementary code [file EMS199501-supplement-Supplementary_code.zip › closingthegap-main/README/complex.png]
