# Supplementary material for: Closing the Gap between Experiment and Simulation—A Holistic Study on the Complexation of Small Interfering RNAs with Polyethylenimine
Source: Mol Pharm. Author manuscript; Available in PMC 2024 Oct 29. (PMC7616749; doi:10.1021/acs.molpharmaceut.3c00747)
Supplement: Supplementary information [file EMS199501-supplement-Supplementary_information.pdf]

Supplementary Information

Closing the gap between experiment and simulation – a holistic study on complexation of small interfering RNAs with polyethyleneimine

Jonas Binder[1], Joshua Winkeljann[2,3], Katharina Steinegger[1], Lara Trnovec[1], Daria Orekhova[1,2], Jonas Zähringer[1,2], Andreas Hörner[3], Valentin Fell[1], Philip Tinnefeld[1,2], Benjamin Winkeljann[1,2*], Wolfgang Frieß[1], and Olivia M. Merkel[1,2,]

[1] Faculty for Chemistry and Pharmacy, Ludwig-Maximilians-Universität München, Butenandtstraße 5-13, Haus B, 81377 München, Germany

[2] Center for NanoScience (CeNS), Ludwig-Maximilians-Universität München, München 80799, Germany

[3] Chair of Experimental Physics I, University of Augsburg, Universitätsstraße 1, 86519 Augsburg, Germany

*****_corresponding author_
Dr. Benjamin Winkeljann
Department of Pharmacy
Ludwig-Maximilians-Universität Munich
Butenandtstraße 5-13, Haus B
81377 München
Germany

e-mail: benjamin.winkeljann@cup.lmu.de
phone: +49 89 218077724

keywords: polyplex, RNA interference, rationale design, MARTINI 3

1 Parametrisation of polyethyleneimine *via* fragmentation

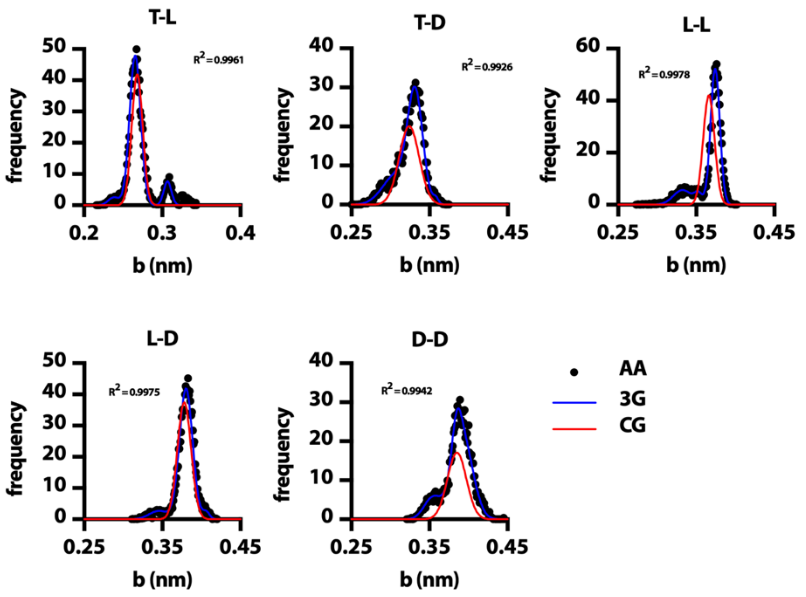

Supplementary Figure 1: Bond fragments of the PEI67 molecule and fit of corresponding AA-distributions through with a 3-peak Gaussian function to extract bonded parameters for CG simulations.

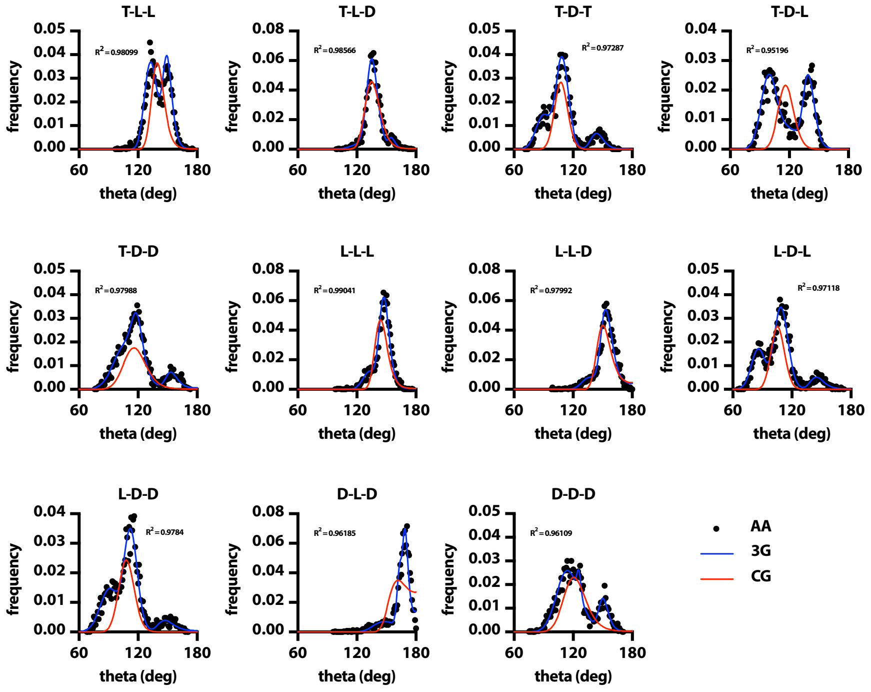

Supplementary Figure 2: Angle fragments of the PEI67 molecule and fit of corresponding AA-distributions through with a 3-peak Gaussian function to extract bonded parameters for CG simulations.

2 Validation using four model PEIs

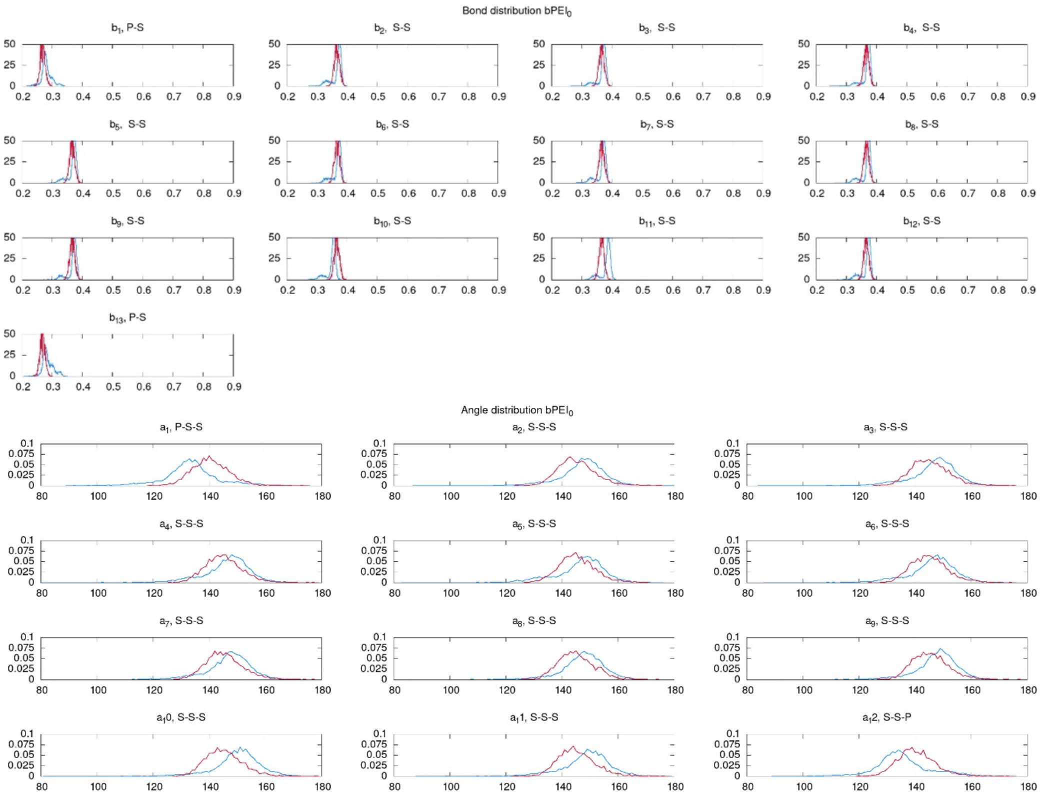

Supplementary Figure 3: Bond and angle distribution of the $bPEI_0$ molecule

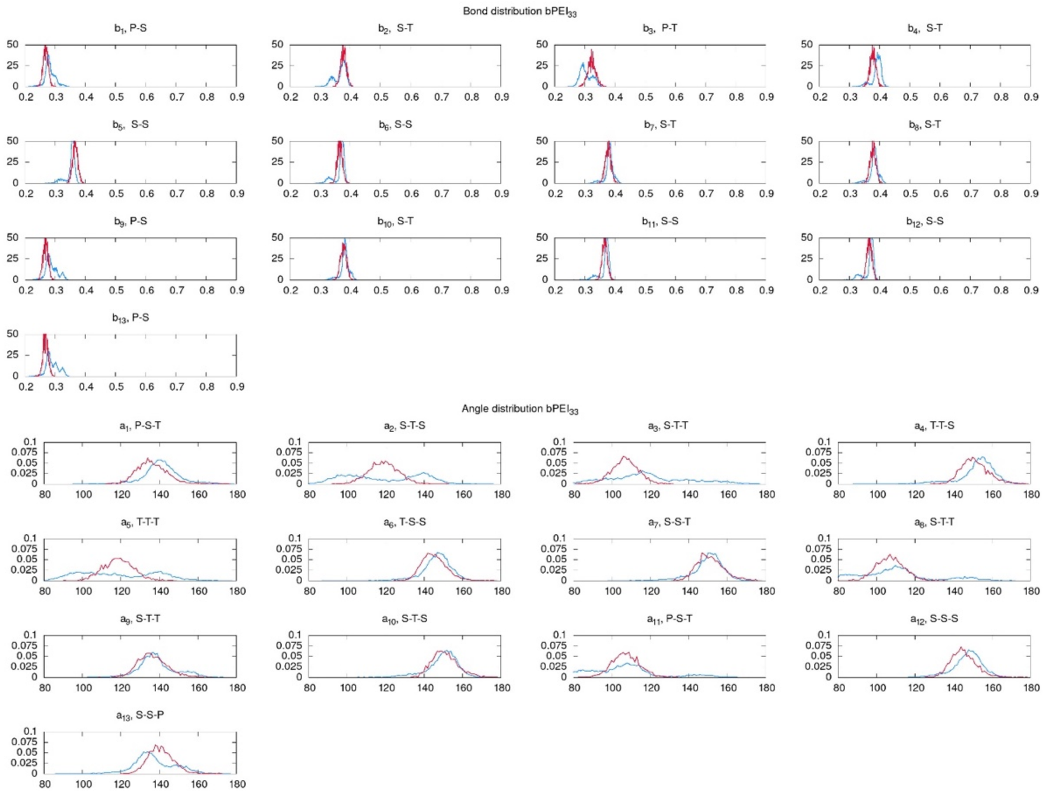

Supplementary Figure 4: Bond and angle distribution of the bPEI$_{33}$ molecule

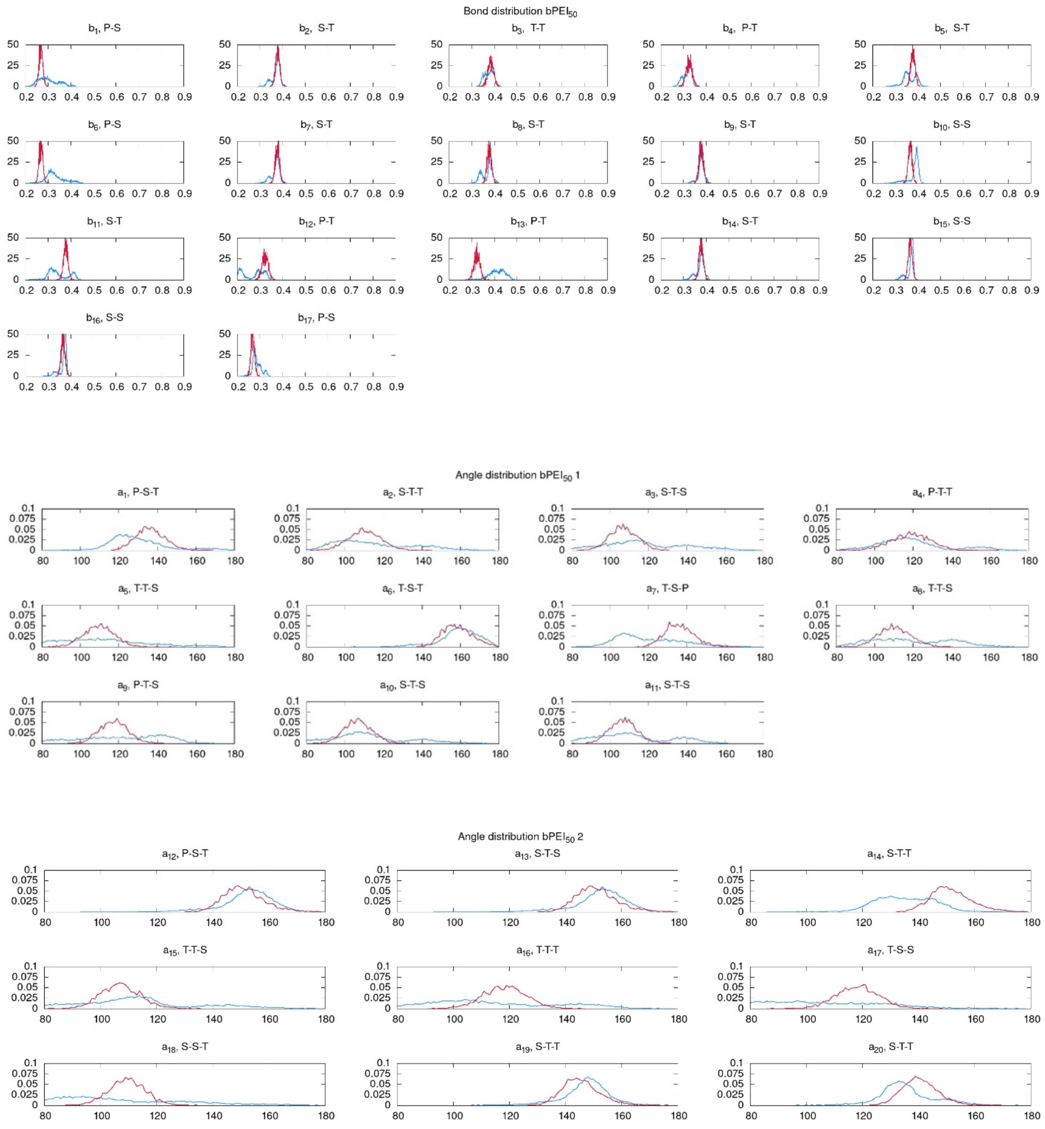

Supplementary Figure 5: Bond and angle distribution of the $bPEI_{50}$ molecule

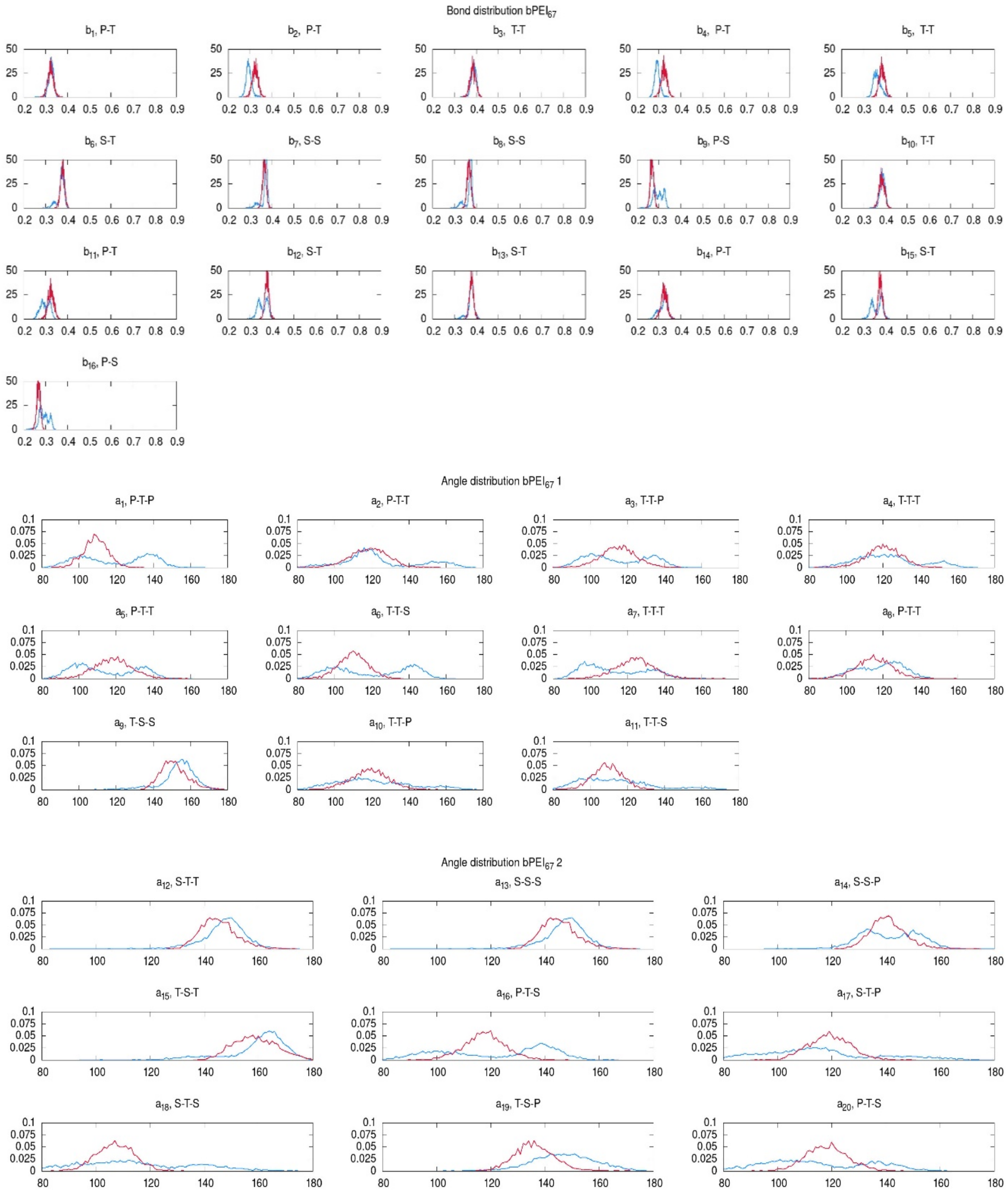

Supplementary Figure 6: Bond and angle distribution of the bPEI$_{67}$ molecule

3 General Program Principle of *The Generator*

• The program starts with a linear bead in tree level 1, which has 2 open binding options.

• Bindings are performed depending on the pre-defined probabilities of *terminatingProbability* and *degreeOfBranching*. The two beads bound to it are assigned to tree level 2.

• Before each new tree level, the maximum polymer mass is compared with the current polymer mass (-> *remainingMass*). In addition, the number of currently open binding options is multiplied by the mass of the terminating bead (-> *terminatingMass*) to determine if polymerization termination needs to be initiated. If this is the case, all remaining binding options are connected to terminating beads and the polymerization ends.

if (remainingMass < terminatingMass):
initiate polymerization termination
else:
proceed to the next tree level

• The open binding options of the two new beads are again occupied depending on the probabilities and the new beads are assigned to tree level 3. This process continues successively until termination is initiated according to the above-mentioned condition.

• After polymerization termination, some calculations are performed on the beads, such as coordinates, bond lengths, and angles.

• Finally, it is checked whether there are directly overlapping beads in all 3 spatial dimensions (*i.e.*, same *x*, *y* AND *z*). If this is the case, the polymerization starts again, otherwise it is successfully completed.

• The SMILES string and its corresponding mapping are then calculated.

• The script completes after generating the output files:

1. itp-file
2. gro-file
3. mapBead-file
4. map-file
5. smiles-file (AA)
6. itp-File for Titratable Martini simulations

Github repository link: https://github.com/jonbind/closingthegap

4 Density Functional Theory

Supplementary Table 1: pK_a and pK_b values at 25°C of different amine groups in the PEI fragment as predicted by Density Functional Theory

Amine	Substitution	pK_a	pK_b
#35	Primary	7.87	6.13
#68	Secondary	8.56	5.44
#76	Tertiary	9.07	4.93
#56	Tertiary	8.60	5.40

Tertiary amines #76 and #56 exhibit decently different pK_b values; the value of #56 is higher than the one of #76, indicating that its protonation is hampered due to its higher sterical shielding compared to #76.

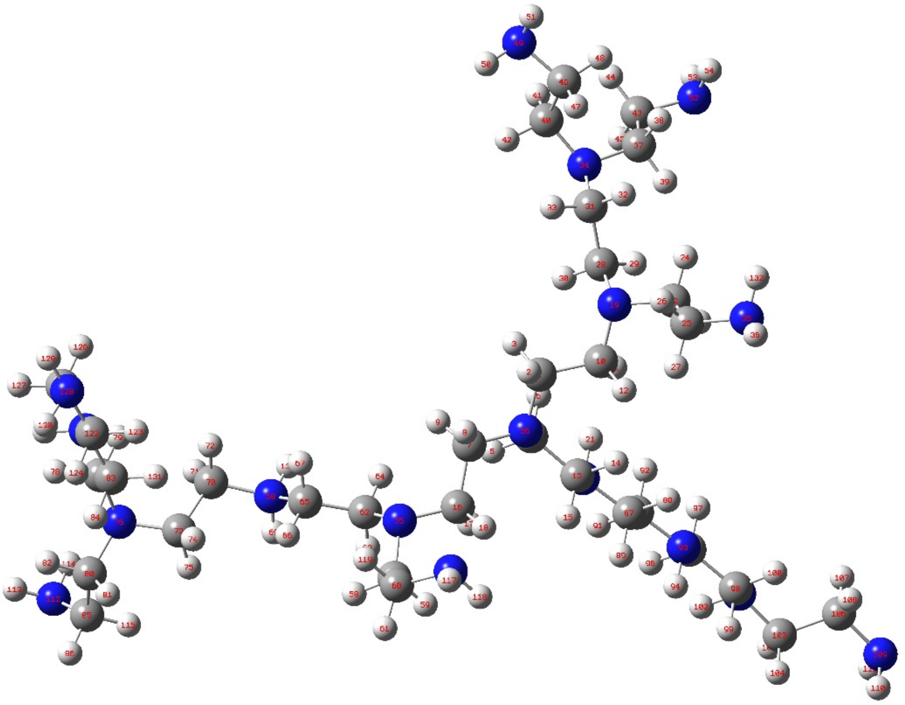

Supplementary Figure 7: PEI model (bPEI67, see main article for details) showing atom labels, the nitrogen atom #68 is here protonated.

5 Metadynamics Simulations

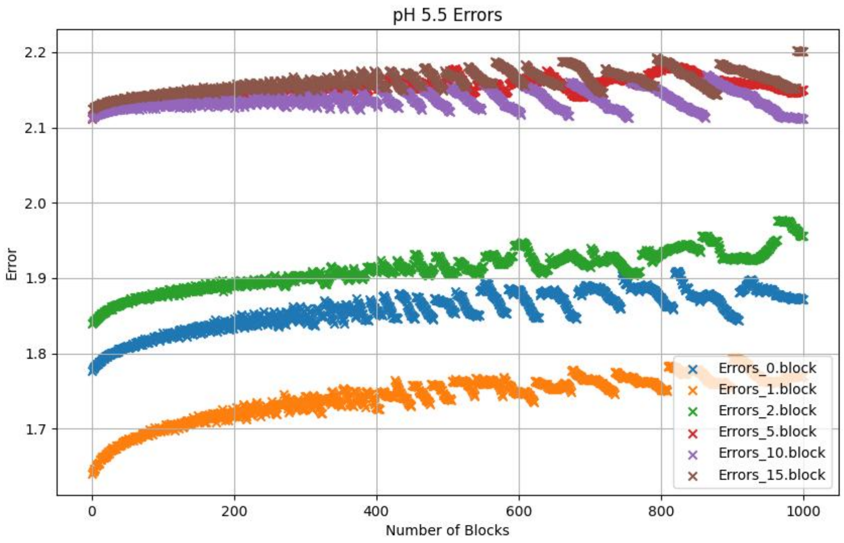

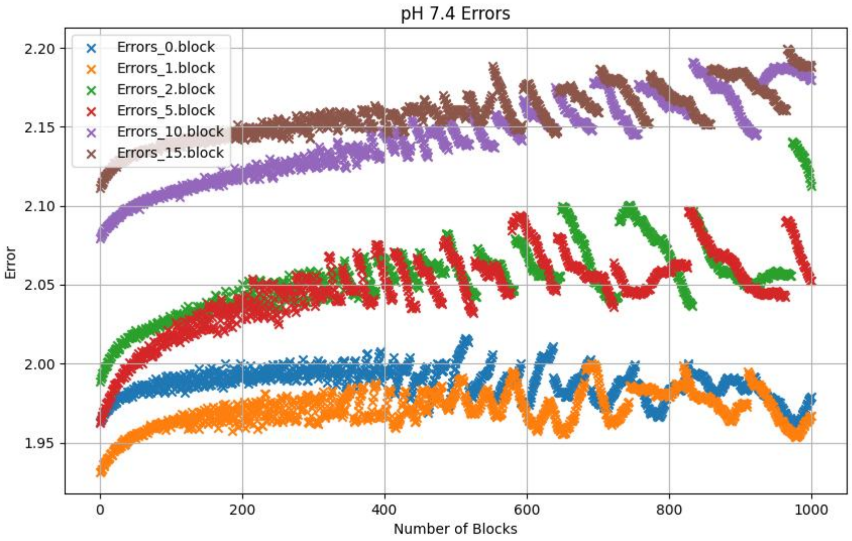

Supplementary Figure 8: Error progression across blocks as conducted using the Plumed block analysis method.

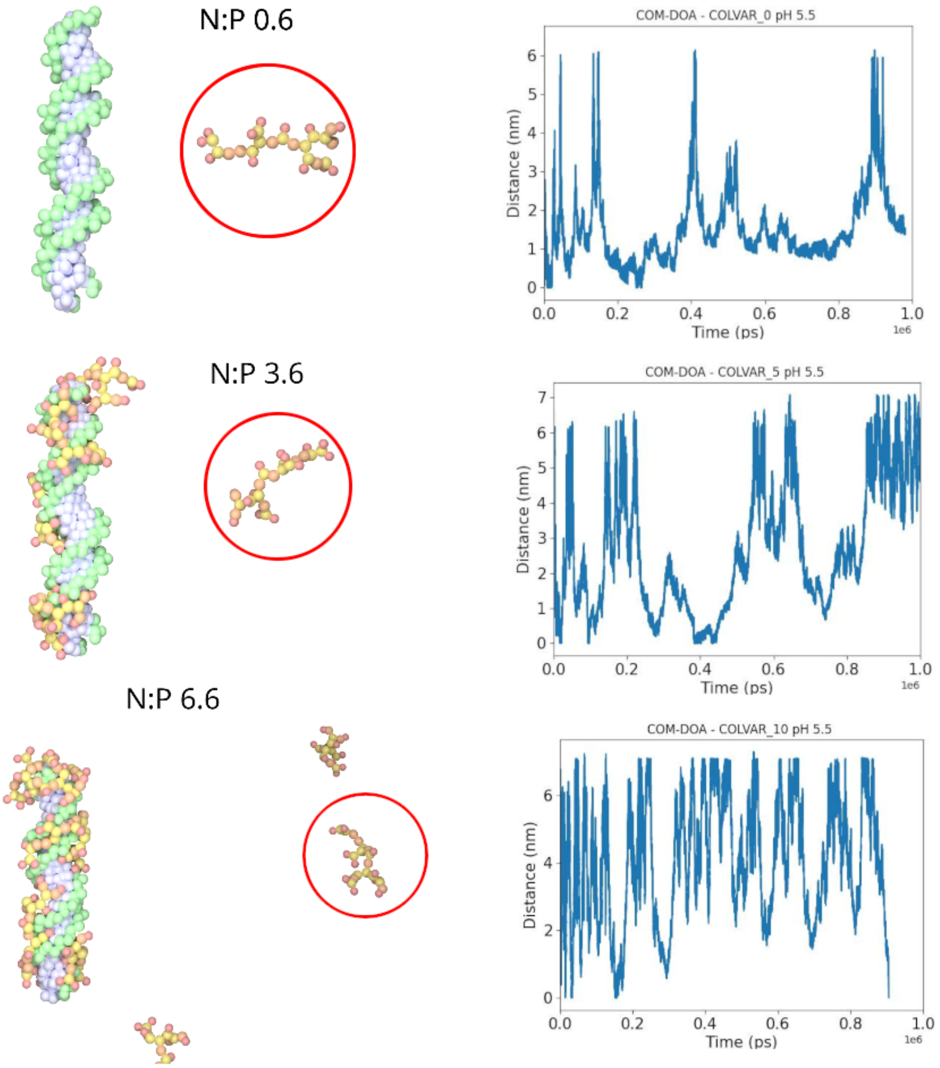

Supplementary Figure 9: State fluctuations of a 1.3 kDa PEI molecule (encircled in red) as it alternates between binding and unbinding to an siRNA molecule at increasing N/P ratios.

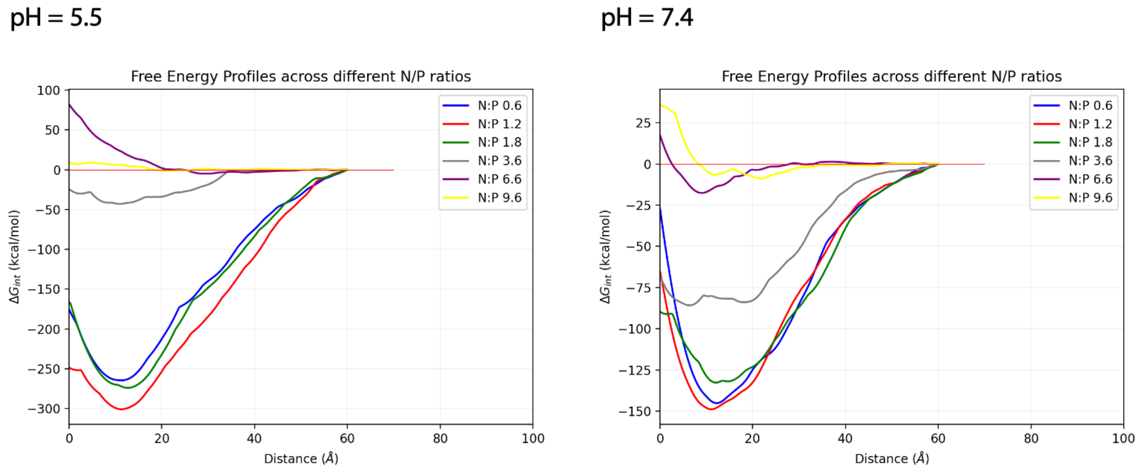

Supplementary Figure 10: Free Energy profiles of individual PEI molecules upon retraction from an siRNA molecule for increasing N/P ratios.
